# Sexually dimorphic gene expression and transcriptome evolution provide mixed evidence for a fast‐Z effect in *Heliconius*

**DOI:** 10.1111/jeb.13410

**Published:** 2019-01-11

**Authors:** Ana Pinharanda, Marjolaine Rousselle, Simon H. Martin, Joe J. Hanly, John W. Davey, Sujai Kumar, Nicolas Galtier, Chris D. Jiggins

**Affiliations:** ^1^ Department of Zoology University of Cambridge Cambridge UK; ^2^ Department of Ecology and Evolutionary Biology and Lewis‐Sigler Institute for Integrative Genomics Princeton University Princeton New Jersey; ^3^ UMR 5554 Institut des Sciences de l‘Evolution CNRS IRD EPHE Université de Montpellier Montpellier France; ^4^ Department of Biology University of York York UK; ^5^ Institute of Evolutionary Biology University of Edinburgh Edinburgh UK

**Keywords:** gene expression, genomics, insects, molecular evolution, sex chromosomes

## Abstract

Sex chromosomes have different evolutionary properties compared to autosomes due to their hemizygous nature. In particular, recessive mutations are more readily exposed to selection, which can lead to faster rates of molecular evolution. Here, we report patterns of gene expression and molecular evolution for a group of butterflies. First, we improve the completeness of the *Heliconius melpomene* reference annotation, a neotropical butterfly with a ZW sex determination system. Then, we analyse RNA from male and female whole abdomens and sequence female ovary and gut tissue to identify sex‐ and tissue‐specific gene expression profiles in *H. melpomene*. Using these expression profiles, we compare (a) sequence divergence and polymorphism; (b) the strength of positive and negative selection; and (c) rates of adaptive evolution, for Z and autosomal genes between two species of *Heliconius* butterflies, *H. melpomene* and *H. erato*. We show that the rate of adaptive substitutions is higher for Z than autosomal genes, but contrary to expectation, it is also higher for male‐biased than female‐biased genes. Additionally, we find no significant increase in the rate of adaptive evolution or purifying selection on genes expressed in ovary tissue, a heterogametic‐specific tissue. Our results contribute to a growing body of literature from other ZW systems that also provide mixed evidence for a fast‐Z effect where hemizygosity influences the rate of adaptive substitutions.

## 1 INTRODUCTION

Heteromorphic sex chromosomes have different evolutionary properties compared to autosomes (Rice, 1984). Specifically, because recessive mutations are exposed to selection more readily on the sex chromosomes, positive selection and purifying selection—as well as the strength of genetic drift—are expected to result in different rates of molecular evolution between sex chromosomes and autosomes. An increased evolutionary rate of sex chromosomes relative to autosomes, known as the fast‐X effect (Charlesworth, Coyne, & Barton, 1987), has been observed in *Drosophila* (e.g., Avila et al., 2014). X genes are expected to diverge faster between species than autosomal genes mainly due to the higher substitution rate of recessive, advantageous mutations. However, this process is also influenced by (a) patterns of selection in males versus females; (b) mutation; (c) recombination; and (d) demography (Connallon, Singh, & Clark, 2012; Kirkpatrick & Hall, 2004; Orr, 2010; Orr & Betancourt, 2001; Pool & Nielsen, 2007; Vicoso & Charlesworth, 2006, 2009).

Patterns of molecular evolution on sex chromosomes are particularly influenced by gene expression patterns. Sexually dimorphic expression is often caused by natural and/or sexual selection favouring phenotypes that influence the fitness of one of the sexes (Grath & Parsch, 2016). In species with genetic sex determination, the majority of sexually dimorphic traits results from the differential expression of genes present in both male and female genomes (Ellegren & Parsch, 2007). Sex‐biased expression is common across taxa from mammals (Rinn & Snyder, 2005) to Diptera (Assis, Zhou, & Bachtrog, 2012), reptiles (Cox et al., 2017), birds (Mank, Nam, & Ellegren, 2010; Mank, Vicoso, Berlin, & Charlesworth, 2010) and Lepidoptera (Rousselle, Faivre, Ballenghien, Galtier, & Nabholz, 2016). For example, in *Drosophila melanogaster*, 57% of genes have been categorized as sex biased (Assis et al., 2012), and, in *Heliconius melpomene*, analysis of two different tissues identified up to 29% of expressed genes as sex biased (Walters, Hardcastle, & Jiggins, 2015). The vast majority of genes that exhibit sexually dimorphic expression are active in reproductive tissues and tend to also have distinctive rates of molecular evolution compared to genes without dimorphic expression (Avila, Campos, & Charlesworth, 2015; Parisi et al., 2003, 2004). Ultimately, the identification of sex‐biased genes, and subsequent analysis of patterns of molecular evolution, will contribute to a better understanding of the evolutionary forces shaping sex chromosome and autosome evolution (Assis et al., 2012; Kirkpatrick & Hall, 2004; Zhang, Hambuch, & Parsch, 2004).

Empirical studies of the fast‐X effect typically measure two different metrics: (a) the ratio of nonsynonymous to synonymous substitution rates (dN/dS) and (b) the amount of adaptive evolution (α) using the McDonald–Kreitman (MK) test (McDonald & Kreitman, 1991). Studies measuring dN/dS usually test for “faster‐X divergence.” Although this approach may be useful for comparing sex chromosome and autosomal divergence, measuring the relative rate of nonsynonymous substitutions captures the effects of both adaptive and neutral (or slightly deleterious) mutations. Estimates of α can better test for an excess of adaptive substitution in the sex chromosome (“faster‐X adaptation”) by combining measures of within‐species polymorphism and between‐species divergence, but α is still sensitive to the rate of accumulation of slightly deleterious mutations and demography (Fay, 2011). For instance, an increase in *N*_e_ is expected to result in decreased dN/dS and increased α even when the rate of adaptive substitutions remains unchanged. To overcome this problem, extensions of the MK test such as ω_a_ were developed to estimate the rate of adaptation by calculating the frequency distribution of polymorphism after correcting for demographic history and distribution of deleterious effects at functional sites (Galtier, 2016).

The analysis of evolutionary rates between sex and autosomal genes, however, has produced mixed evidence in support of fast‐X evolution (Meisel & Connallon, 2013). In some taxa, there is strong evidence for faster‐X divergence but not faster‐X adaptation, or vice versa (Meisel, Malone, & Clark, 2013). For example, the first calculations of faster‐X divergence were carried out in *Drosophila* where support for elevated dN/dS in X genes has been mixed. Studies that used autosome‐to‐X translocations to control for gene content effect did not reach a consensus on the existence of faster‐X divergence (Counterman, Ortiz‐Barrientos, & Noor, 2004; Thornton, Bachtrog, & Andolfatto, 2006; Zhou & Bachtrog, 2012) but X‐linked duplicate genes have elevated dN/dS compared to autosomal duplicates (Thornton & Long, 2002). Signals of faster‐X sequence divergence in *Drosophila* have been shown to affect noncoding regulatory regions as well, and might be at least partly explained by differences in gene composition on the X versus the autosomes (Hu, Eisen, Thornton, & Andolfatto, 2013). However, faster‐X divergence in other taxa has received stronger support. For example, in humans, chimpanzees and rodents, dN/dS is higher for X genes (Mank, Vicoso et al., 2010; Nielsen et al., 2005).

In contrast, whole‐genome analyses of adaptive substitutions have resulted in stronger evidence for faster‐X adaptation in *Drosophila* (Mackay et al., 2012), whereas support for faster‐X adaptation in vertebrates is less clear. McDonald–Kreitman tests support faster‐X adaptation in wild mouse populations (Baines & Harr, 2007) but, for the European rabbit (*Oryctolagus cuniculus*), a clear faster‐X adaptation signal is only present in populations with large effective population sizes (Carneiro et al., 2012).

Taxa with ZW sex determination provide an interesting contrast. For female heterogametic taxa such as birds, females only have one copy of the Z chromosome. A fast‐Z effect may be expected to result from the expression of recessive mutations on the Z chromosome as Z genes are immediately exposed to selection in females (Charlesworth, 2012). In birds, fast‐Z divergence has been reported, but Z male‐biased genes were not less accelerated than unbiased genes or female‐biased genes (Wright, Zimmer, Harrison, & Mank, 2015). This would not be expected if the fast‐Z effect was driven by recessive beneficial mutations, and so, it was suggested that fast‐Z in birds does not reflect positive selection (Mank, Nam, et al., 2010; Wright et al., 2015).

For Lepidoptera, results have also been mixed. Sackton et al. (2014) reported that faster‐Z evolution was driven by positive selection in silkworms. But, in satyrine butterflies, there were no significant differences in adaptive evolutionary rates between the Z and the autosomes (i.e., no fast‐Z adaptation). However, the comparison of male‐biased, female‐biased and unbiased Z genes in satyrine butterflies revealed increased purifying selection against recessive deleterious mutations in female‐biased Z genes (Rousselle et al., 2016). Therefore, considerable uncertainty remains regarding the prevalence and magnitude of the fast‐X/Z effect on divergence and adaptation.

Here, we investigate the effects of hemizygosity on the rates of adaptive substitution in the neotropical butterfly genus *Heliconius,* a ZW sex determination system, by analysing polymorphism, divergence and gene expression genomewide. We test whether there is a fast‐Z effect in *Heliconius* using two species from the *H. melpomene* and *H. erato* clades which diverged 12 million years ago (synonymous divergence = 0.15) (Kozak et al., 2015; Martin et al., 2016). Previous analyses of *Heliconius* transcriptome data have focused on the evolution of dosage compensation and the impact of sex‐specific dosage on the levels of gene expression (Walters et al., 2015). In this study, using the same transcriptome data, we first compute sex‐biased expression. Then, accounting for sex‐biased gene expression, we (a) calculate coding sequence divergence and polymorphism in *H. melpomene* and (b) assess the strength of positive and negative selection, and rates of adaptive evolution between *H. melpomene* and *H. erato*. We then analyse newly generated female transcriptome data from *H. melpomene* ovary and gut tissue in order to investigate whether genes expressed in the reproductive tissue of the heterogametic sex have higher rates of adaptive evolution than those expressed in somatic tissues.

## 2 MATERIALS AND METHODS

### 2.1 Updated *H. melpomene* annotation

The Hmel2 annotation of the *H. melpomene* genome has 13,178 predicted transcripts spanning 16,897,139 bp (Davey et al., 2016 The Heliconius Genome Consortium, 2012). The Hmel2 annotation is incomplete, as there are 20,118 high‐quality predicted transcripts in *H. erato* spanning 33,669,374 bp (Van Belleghem et al., 2018). To improve the completeness of the annotation for *H. melpomene,* we downloaded RNA‐seq reads from NCBI repositories ArrayExpress ID: E‐TAB‐1500 (Briscoe et al., 2013) and BioProject PRJNA283415 (Walters et al., 2015), published since Hmel1 release. We also used data from 10 wing RNA‐seq libraries (Hanly, 2017). We used the BRAKER1 pipeline to perform unsupervised RNA‐seq‐based genome annotation (Hoff, Lange, Lomsadze, Borodovsky, & Stanke, 2016). GeneMark‐ET was used to perform iterative training, generating initial gene structures, and AUGUSTUS was used for training and subsequent integration of RNA‐seq read information into the final gene predictions (Hoff et al., 2016 ; Lomsadze, Burns, & Borodovsky, 2014; Stanke , Diekhans, Baertsch, & Haussler, 2008). This resulted in 26,017 predicted transcripts spanning 32,222,367 bp. 6,532 of these transcripts had a 90% single hit match to the Repbase repeat database. We considered the 6,532 transcripts to be repeat proteins and removed them (Bao, Kojima, & Kohany, 2015). We transferred 428 manually annotated genes (441 transcripts/protein) from the original Hmel2 annotation and removed any BRAKER1 predictions that overlapped. We also transferred 189 genes (189 transcripts/proteins) that have been manually annotated and published since Hmel2 release. Specifically, we transferred 73 gustatory receptors, 31 immune response and 85 glutathione‐S‐transferases and glucuronosyltransferases (Briscoe et al., 2013; van Schooten, Jiggins, Briscoe, & Papa, 2016; Yu, Fang, Zhang, & Jiggins, 2016) and removed any BRAKER1 predictions that were overlapping. Moreover, BRAKER1 predictions that had 1‐to‐1 overlaps with Hmel2 names were replaced by their original Hmel2 name. For many‐to‐1 mapping between the BRAKER1 predictions and Hmel2, Hmel2 names were reused and a suffix of g1/g2/g3/etc. was added. The rest were renamed from HMEL030000 onwards.

### 2.2 Samples for gene expression analysis

Gene expression data were calculated using (a) Illumina 100‐bp paired‐end RNA‐seq data from five Panamanian *H. m. rosina* whole‐male abdomens, and five Panamanian *H. m. rosina* whole‐female abdomens, downloaded from GenBank (BioProject PRJNA283415) (Walters et al., 2015) and (b) newly sequenced Illumina HiSeq 2500 150‐bp paired‐end directional (stranded) RNA‐seq data from ovary tissue of seven young (1 hr) and six old (20 days) *H. m. rosina* females and from gut tissue of six young (1 hr) and six old (20 days) *H. m. rosina* females (25 samples from 13 different individuals, Supporting Information Table S1).

For these 25 samples, *H. m. rosina* females were reared in insectaries in Gamboa, Panama. *Passiflora platyloba* potted plants were monitored daily and 5th‐instar caterpillars were removed and taken to the laboratory in large individual containers where they were allowed to pupate and emerge at a constant temperature (24–25°C). The pupating containers in the laboratory were monitored several times a day. Sexually antagonistic pressures are expected to be greater in mature adults, and genes exhibiting extreme sexually dimorphic patterns should be expressed in mature individuals (Gibson, Chippindale, & Rice, 2002). In addition, mating induces behavioural and physiological changes and has been shown to trigger regulatory changes in sex‐biased genes (e.g., Immonen, Sayadi, Bayram, & Arnqvist, 2017). By collecting gene expression data from ovaries at two different life‐history time points, we aimed to increase the number of sex‐specific genes identified. When a female emerged, it was either (a) returned to the insectaries to be mated to a *H. m. rosina* male (Treatment: old, Supporting Information Table S1) or (b) dissected 1 hr after eclosion under controlled laboratory conditions (Treatment: young, Supporting Information Table S1). Mated females were kept in individual 1 m × 1 m × 2 m cages for 20 days until dissection.

Guts and ovaries were dissected in RNAlater (ThermoFisher, Waltham, MA) at 24–25°C, and tissue was stored in RNAlater at 4°C for 24 hr and −20°C thereafter. Total RNA was extracted with a combined guanidium thiocyanate–phenol–chloroform and silica matrix protocol using TRIzol (Invitrogen, Carlsbad, CA), RNeasy columns (Qiagen, Valencia, CA) and DNaseI (Ambion, Naugatuck, CT). mRNA was isolated from total RNA via poly‐A pull‐down, and directional cDNA library preparation and sequencing (Illumina HiSeq 2500, 150‐bp paired end) were performed by Novogene Bioinformatics Technologies (Hong Kong, China) (Supporting Information Table S1).

### 2.3 Read mapping, counting and identification of sex‐ and ovary‐ and gut‐biased genes

FASTQ reads were aligned to gene sequences from *H. melpomene* v2.5 annotation using HISAT2 (Kim, Langmead, & Salzberg, 2015) with default mapping parameters. Mapping statistics were calculated using samtools flagstat (v1.2) (Li et al., 2009). We used htseq‐count to determine the number of aligned sequencing reads mapped to each genic feature (htseq v0.6.1; python v2.7.10; option: ‐m union) (Anders, Pyl, & Huber, 2015).

Estimation of variance–mean dependence from the count data was performed with deseq2 (v1.14.1) (Love, Huber, & Anders, 2014) using bioconductor v3.4 and r v3.2.5, using the constructor function DESeqDataSetFromHTSeqCount(design = ~batch + tissue) for ovary‐ and gut‐biased genes. All the result tables were built using the DESeq2 results() function (options: betaPrior = false, test = Wald). We filtered the results as in Walters et al. (2015) with FDR < 0.05 (alpha = 0.05) (Walters et al., 2015). Ovary‐ and gut‐biased genes have a log_2_‐fold change significance threshold >1.5 (option: lcfThreshold < 1.5, altHypothesis = “greaterAbs”). We defined male, female and unbiased genes as in Rousselle et al. (2016). First, we calculated reads per kilobase per million (RPKM) as:

$$\text{RPKM}=\frac{N_{c}\times 10^{9}}{N_{\mathrm{tot}}\times L_{c}}$$

where *N*_c_ is the number of reads mapped to the genic feature, *N*_tot_ is the total number of reads mapped in the sample, and *L*_c_ is the length of the genic sequence in base pairs (Mortazavi, Williams, McCue, Schaeffer, & Wold, 2008). RPKM_*i*_ is the mean RPKM of gene *i* across the 10 individuals. Genes for which RPKM_f_/RPKM_m_ _ _> 1.5 were classified as female biased, genes for which RPKM_f_/RPKM_m _ < 0.66 were classified as male biased, and the others were classified as unbiased (Rousselle et al., 2016).

### 2.4 Extraction of orthologous genes, coding sequence alignment and SNP calling

OrthoFinder was used to identify orthologous groups of genes in the *H. melpomene* and the *H. erato* transcriptomes (options: ‐t 48 ‐a 6). 1‐1 orthologous gene sequences were selected for use in subsequent analysis (Supporting Information Table S2). Using Gff‐Ex, a genome feature extraction package (Rastogi & Gupta, 2014), we extracted coding sequences from (a) 10 whole‐genome short‐read resequenced wild *H. m. rosina* from Panama (Supporting Information Table S3; Van Belleghem et al., 2018) mapped to Hmel2 (Davey et al., 2016) with bwa‐mem (Li & Durbin, 2009) and (b) the reference *H. erato* genome (Van Belleghem et al., 2018).

For the 10 whole‐genome resequence *H. m. rosina* samples (Van Belleghem et al., 2018), genotypes were called using HaplotypeCaller (GATK v3.4‐0‐g7e26428) (DePristo et al., 2011), and genotypes were designated as missing if the read depth for a given individual at a given site was <8. Coding sequences for 1‐1 orthologous genes were extracted in fasta format from (a) and (b) and aligned using MACSE, accounting for frameshifts and stop codons (Ranwez, Harispe, Delsuc, & Douzery, 2011).

### 2.5 Calculation of diversity and selection statistics for 1‐1 ortholog alignments between *H. melpomene* and *H. erato*:* Classic approach*

The adaptive substitution rate was estimated by comparing synonymous and nonsynonymous variation in the polymorphism and divergence compartments, as first proposed by McDonald & Kreitman, 1991; see also Bustamante et al., 2005, and Macpherson, Sella, Davis, & Petrov, 2007). We first used the original MK test (referred to as *Classic approach* hereafter) to estimate the rate of adaptive substitution for all genes found to be orthologous between *H. melpomene* and *H. erato*. We calculated (a) synonymous polymorphism (*P*_s_) and (b) nonsynonymous polymorphism (*P*_n_) in *H. melpomene,* as well as (c) synonymous fixed divergence (dS), and (d) nonsynonymous fixed divergence (dN) between *H. melpomene* and *H. erato*. We estimated the rate of adaptive molecular evolution (α) between the two species as:

$$\alpha =1-\frac{\text{dS}\;\times \;P_{n}}{\text{dN}\;\times \;P_{s}}$$

α assumes that nonsynonymous mutations are either adaptive, neutral or strongly deleterious (McDonald & Kreitman, 1991), with −∞ > α ≥ 1, where α = 0 represents the null hypothesis that nonsynonymous mutations are neutral (dN/dS = *P*_n_/*P*_S_). α > 0 corresponds to dN/dS > *P*_n_/*P*_S_ and indicates positive selection, whereas α < 0 corresponds to dN/dS < *P*_n_/*P*_S_ and indicates negative selection. These values were calculated using the EggLib C++ function polymorphismBPP (v2.1.11) (De Mita & Siol, 2012) and Bio++ (v2.2.0) (Dutheil & Boussau, 2008) in python (v2.7.5) using scripts adapted from https://github.com/tatumdmortimer (last accessed 09/04/2018) (O'Neill, Mortimer, & Pepperell, 2015).

### 2.6 Calculation of diversity and selection statistics for 1‐1 ortholog alignments between *H. melpomene* and *H. erato*:* Modelling approach*

The *Classic approach* to the MK test is robust to differences in mutation rates and variation in coalescent histories across genomic locations (McDonald & Kreitman, 1991). Inference of positive selection using the *Classic approach* of the MK test is not robust, however, to the occurrence of slightly deleterious mutations and demographic change. To account for these confounders, we used a *Modelling* approach to estimate the strength of positive and purifying selection in addition to the *Classic approach* described above, using the method of Eyre‐Walker and Keightley (2009) as implemented in Galtier (2016) and Rousselle et al. (2016).

The *Modelling approach* uses the frequency distribution of polymorphism to assess the distribution of deleterious mutations at functional sites. This elaborates on the *Classic approach* of the MK test by modelling the distribution of fitness effects (DFE) of deleterious nonsynonymous mutations as a negative Gamma distribution. The model is fitted to the synonymous and nonsynonymous site frequency spectra (SFS) and the expected dN/dS under near‐neutrality is inferred. The difference between the observed and expected dN/dS provides an estimate of the proportion of adaptive nonsynonymous substitutions (α). The per‐mutation rate of adaptive substitutions is calculated as:

$$\omega_{a}=\;\alpha \;\times \;\frac{\text{dN}}{\text{dS}}$$

and the per‐mutation rate of nonadaptive substitutions is calculated as:

$$\omega_{\mathrm{na}}=\left(1\;-\alpha \right)\;\times \;\frac{\text{dN}}{\text{dS}}$$

### 2.7 Gene expression level and π_n_/π_s_ ratios

To test whether gene expression level and chromosome type have a significant effect on π_n_/π_s_ ratios, we used a multiple regression analysis. We established the linear model:

$$\log\left(\pi_{\mathrm{nij}}\right)∼\log\left(\pi_{\mathrm{nsj}}\right)+\text{chromosome}\_{\text{type}}_{j}+\text{log}\left(\text{RPKM}\right)$$

using r (v3.2.5). Chromosome type is either autosome or sex chromosome as assigned in Hmel2 reference genome (Davey et al., 2016). 477 genes with no polymorphism were removed from the analysis. We plotted diagnostic plots of residuals versus fitted values.

## 3 RESULTS

### 3.1 Hmel2.5 annotation and 1‐1 ortholog prediction with *H. erato*

There are 20,118 transcripts predicted in *H. erato* (Van Belleghem et al., 2018) and 20,097 genes (21,565 transcripts/proteins) in *H. melpomene* Hmel2.5. OrthoFinder returned 11,062 clusters of genes, 8085 of which included exactly one sequence per species. 14,841 (73.8%) of the total number of genes in *H. erato* were assigned to an orthogroup, and 14,857 (68.6%) of the total number of genes in *H. melpomene* version Hmel2.5 were assigned to an orthogroup (Supporting Information Table S2). Conversely, the *H. melpomene* Hmel2 annotation has 13,019 predicted gene models (Davey et al., 2016). Using the Hmel2 annotation, OrthoFinder returned 9,320 clusters of genes, 6,846 of which included exactly one sequence per species (i.e., single‐copy orthogroups). 13,744 (68.3%) genes were assigned to an orthogroup in *H. erato* and 10,530 (80.9%) were assigned to an orthogroup in *H. melpomene*. The Hmel2.5 annotation set for *H. melpomene* is therefore more comparable to the published *H. erato* gene annotation and is more appropriate for future gene‐based analysis in *H. melpomene*. The Hmel2.5 annotation has 1,093 genes mapping to the Z chromosome and 18,835 mapping to the autosomes. This new version of *H. melpomene* genome annotation was numbered Hmel2.5 (available at LepBase http://ensembl.lepbase.org/Heliconius_melpomene_melpomene_hmel25/Info/Index, last accessed 20 June 2018, Challis et al., BioRxiv preprint).

### 3.2 RNA‐sequencing and read mapping

Analysis of gene expression profiles in the data retrieved from Walters et al. (2015) by principal component, the first principal component separates gene expression in whole abdomen by sex and explains 97% of variance (Supporting Information Figure S1). The 25 *H. melpomene* samples sequenced for this project have a median total number of reads of 34.86 M (min. 27.81 M; max. 46.12 M), similar to previously published gene expression studies in *Heliconius* (Briscoe et al., 2013; Walters et al., 2015. Mapping success is high compared to other published studies (e.g., Walters et al., 2015; Yu et al., 2016) (Supporting Information Table S1). We analysed data from two different time points and from non‐sex‐ and sex‐specific tissue separately (Treatment: Young and Old). There is a clear separation of the 25 samples by tissue when we compare gene expression profiles between them. In total, 51% of the total variance is explained by the two‐first principal components. PC1 separates the samples by tissue and explains 40% of variance. PC2 explains 11% of total variance and separates samples by age (Supporting Information Figure S3). Ovarian tissue clusters by age more tightly than non‐sex‐specific tissue (Gut) (Supporting Information Figure S4A,B).

In order to test whether there was a fast‐Z effect in *Heliconius,* we calculated the following statistics for autosomal and Z genes: dN/dS, α, ω_a_ and ω_na_. We also calculated π_sZ_/π_sA_ and investigated the relationship between π_n_ and gene expression. dN/dS tests for “faster‐Z divergence.” If there is a faster‐Z effect in a female heterogametic system, genes with female‐biased expression will have higher dN/dS than male‐biased or unbiased genes. α, ω_a_ and ω_na_ test for “faster‐Z adaptation.” In a female heterogametic system, faster‐Z adaptation would predict that the proportion of adaptive nonsynonymous substitutions (α) in female‐biased genes will be significantly higher in the Z chromosome compared to the autosomes. ω_a_ measures the per‐mutation rate of adaptive substitutions and, if there is “fast‐Z adaptation,” ω_a_ is also predicted to be significantly higher for female‐biased Z genes. However, in a female heterogametic system with reduced purifying selection on the Z, the per‐mutation rate of nonadaptive substitution (ω_nα_) would also be higher for female‐biased Z genes. Another metric that can be used as an indicator of the strength of purifying selection is the π_sZ_/π_sA_ ratio. In a female heterogametic system, stronger purifying selection on the Z chromosome would lead to a π_sZ_/π_sA_ ratio <0.75 due to background selection. Overall, patterns of diversity tend to be associated with gene expression levels. π_n_ is expected to be negatively correlated with expression levels if there is increased purifying selection on highly expressed genes.

### 3.3 Coding sequence divergence does not support a significant fast‐Z effect

We first compared rates of Z and autosomal sequence divergence using dN/dS comparisons of 1‐1 orthologous genes between *H. melpomene* and *H. erato*. The dN/dS ratio for the Z chromosome genes is not significantly higher than dNdS for autosomal genes (dN/dS_Auto_ = 0.110; 95% CI = [0.106–0.113]; dN/dS_Z_ = 0.120; 95% CI = [0.098–0.145]), indicating no obvious faster‐Z divergence of coding sequence.

More highly expressed genes are more exposed to selection, so in a female heterogametic system with a fast‐Z effect, genes with female‐biased expression are expected to have higher rates of amino acid substitution if dN/dS is driven by positive selection. However, the dN/dS ratio of Z female‐biased genes (dN/dS_Z_ = 0.120; 95% CI = [0.069–0.183]) was not significantly different to that for male‐biased genes (dN/dS_Z_ = 0.148 [0.122–0.172]) or unbiased genes (dNdS_Z_ = 0.107; 95% CI = [0.078–0.143]). By contrast, among autosomal genes, those that are unbiased have a significantly lower coding sequence divergence compared to both male‐biased and female‐biased autosomal genes (dNdS_Auto_ = 0.0978; 95% CI = [0.093–0.102]) (Table 1).

**Table 1:** Ratios of π_n_/π_s_, dN/dS in *H. melpomene*; and calculations of α, ω_a_ and ω_na_ for autosomal and Z male‐biased, female‐biased and unbiased genes between *H. melpomene* and *H. erato*

	Linkage	All	Female biased	Male biased	Unbiased
π_n_/π_s_	Autosomal	0.103 [0.100–0.107]	0.106 [0.10–0.113]	**0.127** ^**(A)**^ [0.118–0.138]	**0.094** ^**(A)**^ [0.091–0.098]
	Z	0.111 [0.098–0.126]	0.094 [0.059–0.136]	0.136 [0.112–0.162]	0.104 [0.09–0.125]
dN/dS	Autosomal	0.110 [0.106; 0.113]	0.113 [0.105; 0.121]	0.113 [0.145; 0.167]	**0.098** ^**(A)**^ [0.093; 0.102]
	Z	0.120 [0.098–0.145]	0.120 [0.069–0.183]	0.148 [0.122–0.172]	0.107 [0.078–0.143]
α Classic	Autosomal	0.24 [−0.486 to 0.697]	0.12 [−0.54 to 0.687]	0.305 [−0.309 to 0.669]	0.225 [−0.544 to 0.719]
	Z	0.434 [−0.526 to 0.866]	0.463 [−0.276 to 0.837]	0.279 [−0.728 to 0.794]	0.535 [−0.475 to 1.0]
α Modelling	Autosomal	0.629 [0.622–0.636]	0.635 [0.620–0.650]	0.630 [0.616–0.646]	**0.538** ^**(B)**^ [0.529–0.547]
	Z	**0.675** ^**(A)**^ [0.647–0.704]	0.699 [0.595–0.811]	0.646 [0.596–0.697]	**0.537** ^**(B)**^ [0.500–0.576]
ω_a_	Autosomal	0.062 [0.061–0.063]	0.066 [0.065–0.068]	**0.087** ^**(B)**^ [0.085–0.089]	**0.047** ^**(B)**^ [0.046–0.048]
	Z	**0.069** ^**(A)**^ [0.066–0.072]	0.069 [0.058–0.080]	**0.090** ^**(B)**^ [0.083–0.097]	**0.048** ^**(B)**^ [0.044–0.051]
ω_na_	Autosomal	0.036 [0.036–0.037]	0.038 [0.037–0.040]	**0.051** ^**(A)**^ [0.049–0.053]	0.040 [0.039–0.041]
	Z	0.033 [0.030–0.036]	0.029 [0.019–0.040]	0.049 [0.042–0.056]	0.041 [0.038–0.044]
#Genes	Autosomal	7464	1231	1238	4739
	Z	200	28	96	193

Notes

π_n_/π_s_, dN/dS ratios, α, ω_a_ and ω_na_ were calculated for autosomal and Z male‐biased, female‐biased and unbiased genes. π_n_/π_s_, dN/dS ratios were calculated for *H. melpomene* samples, and α, ω_a_ and ω_na_ were calculated between *H. melpomene* and *H. erato*. Intervals represent 95% confidence intervals obtained by bootstrapping 1,000 times. Bold^(A)^ denotes significant values within either Z or autosomal categories. Bold^(B)^ denotes significant values within both Z and autosomal categories. Significance indicated separately for *All* and for sex‐biased expression (*female*,* male* and *unbiased*).

Finally, dS on the Z chromosome (dS_Z_ = 0.189; 95% CI = [0.18–0.2]) is higher than dS on the autosomes (dS_Auto_ = 0.162; 95% CI = [0.16–0.17]), consistent with either a: (a) male‐biased mutation rate or (b) difference in coalescence time for autosomes and Z, but does not support a fast‐Z effect (Table 1).

### 3.4 π_sZ_/π_sA_ diversity ratio is lower than 0.75

Next, we explored patterns of within‐species diversity as an indicator of the strength of purifying selection. In a population at equilibrium with a 1:1 sex ratio, the π_sZ_/π_sA_ diversity ratio is expected to be 0.75, but stronger purifying selection on the Z chromosome would lead to a reduction in this ratio due to background selection. The π_sZ_/π_sA_ ratio for *H. melpomene* is approximately 0.44 (Table 2), which might indicate purifying selection on the Z. However, this ratio can also be influenced by a biased sex ratio (Vicoso & Charlesworth, 2006), differences in recombination rates (Charlesworth, 2012), sex‐biased mutation rates (Vicoso & Charlesworth, 2009) or a historical reduction in population size. Recent calculations for *H. melpomene* from Panama using whole‐genome short‐read sequencing data estimated the π_sZ_/π_sA_ diversity ratio value to be 0.611; CI = [0.570–0.653] with only weak evidence for a population bottleneck (Van Belleghem et al., 2018). The more pronounced reduction in diversity at synonymous sites observed might, therefore, indicate enhanced background selection in genic regions of the Z chromosome.

**Table 2:** *H. melpomene* π_s_ and π_sZ_/π_sA_ ratio from pairwise alignments for Z and autosomal genes

	Linkage	All	Female biased	Male biased	Unbiased
π_s_	Autosomal	**0.027** ^**(A)**^ [0.026–0.027]	0.025 [0.024–0.027]	**0.035** ^**(A)**^ [0.033–0.036]	0.025 [0.024–0.026]
	Z	0.012 [0.011–0.013]	0.016 [0.013–0.020]	0.015 [0.01–0.02]	0.0106 [0.009–0.012]
π_sZ_/π_sA_	NA	0.444	NA	NA	NA

π_s_ calculated from pairwise alignments for Z and autosomal genes. π_sZ_/π_sA_ ratio used to estimate N_eZ_/N_eA_. Intervals represent 95% confidence intervals obtained by bootstrapping genes (1,000 replicates). Bold ^(A)^ denotes significant values within either Z or autosomal categories. Significance indicated separately for *All* and for sex biased (*female*,* male* and *unbiased*).

### 3.5 Increased strength of purifying selection on highly expressed genes

**Figure 1:**
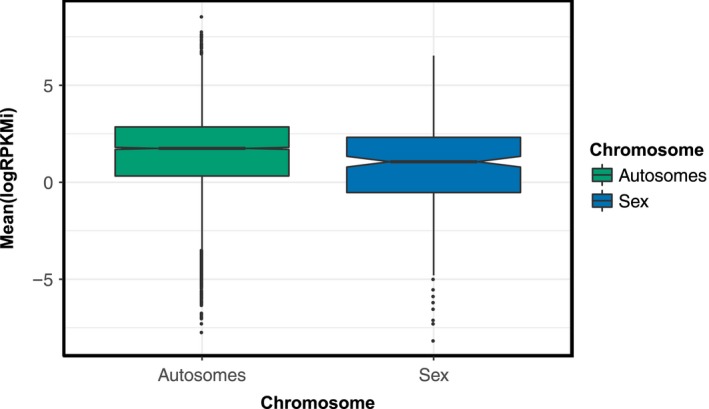
Expression level of Z and autosomal genes. Median expression level of Z genes is significantly lower than autosomal genes (*p *<* *0.05). Notches on boxplot display the confidence intervals around the median

Patterns of diversity were, however, strongly associated with expression levels. Using a multiple regression approach, we found that functional genetic diversity, π_n,_ was significantly negatively correlated with expression level for both autosomal and Z genes (*p *<* *0.01) consistent with increased purifying selection on highly expressed genes (Supporting Information Figure S2) (Figure 1).

### 3.6 Z and autosomal rates of adaptive substitution: testing fast‐Z adaptation

We next explored patterns of adaptive evolution using (a) the *Classic* MK test and (b) the *Modelling* approach which accounts for the effect of mildly deleterious mutations. We computed (a) the proportion of adaptive nonsynonymous substitutions (α) for both the *Classic* and the *Modelling* approaches and (b) ω_a_ and ω_na_ for the *Modelling approach*. ω_a_ is the per‐mutation rate of adaptive substitutions and ω_na_ is the per‐mutation rate of nonadaptive substitutions.

There are no significant differences in α values between Z and autosomal genes under the *Classic approach* (Table 1). However, using the *Modelling approach*, when all genes are considered, Z genes have a marginally but significantly higher α (α_Z_ = 0.675; 95% CI = [0.647–0.704]) than those that are autosomal (α_Auto_ = 0.629; 95% CI = [0.622–0.636]). Nonetheless, α is not significantly different between the Z chromosome and autosomes for female‐biased (α_Auto_ = 0.635; 95% CI = [0.62–0.65]; α_Z_ = 0.699; 95% CI = [0.595–0.811]) or male‐biased genes (α_Auto_ = 0.63; 95% CI = [0.616–0.646]; α_Z_ = 0.646; 95% CI = [0.596–0.697]). Unbiased genes have significantly lower α values than female‐ or male‐biased genes for both Z and autosomes, but within the unbiased genes there is no significant difference in α between Z (α_Z_ = 0.537; 95% CI = [0.5–0.567]) and autosomes (α_Auto_ = 0.538; 95% CI = [0.529–0.547]) (Table 1). This means that the significant effect we find in the Z (α_Z_ = 0.675; 95% CI = [0.647–0.704]) when analysing all the genes together is due to the sex‐biased genes and not due to the unbiased gene category. This observation is in accordance with the predictions from Rice (1984), where the accumulation of alleles under sex‐specific and antagonistic selection on the Z was expected if the alleles were recessive in females and dominant in males (Rice, 1984). The lack of significant differences in α between sex‐biased genes is not consistent with the expectations of fast‐Z adaptation, which would predict faster evolution of female‐biased genes due to hemizygosity compared to autosomes, but this could also reflect a lack of power to detect the signal when the total number of genes is reduced.

### 3.7 Hemizygosity and the rate of adaptive substitutions

There was no evidence for reduced purifying selection on the Z chromosome, as the per‐mutation rate of nonadaptive substitution (ω_nα_) is lower for Z genes (ω_nαZ_ = 0.033; 95% CI = [0.030–0.036] and ω_nα_ _Auto_ = 0.036; 95% CI = [0.036–0.037]). Female‐biased genes have the lowest ω_nα_ (ω_nα_ _Auto_ = 0.038; 95% CI = [0.037–0.040]; ω_nα__Z_ = 0.029; 95% CI = [0.019–0.040]) compared to male‐biased (ω_nα_ _Auto_ = 0.051; 95% CI = [0.049–0.053]; ω_nα__Z_ = 0.049; 95% CI = [0.042–0.056]) and unbiased (ω_nα_ _Auto_ = 0.04; 95% CI = [0.039–0.041]; ω_nα__Z_ = 0.041; 95% CI = [0.038–0.044]) genes, which confirms the low π_n_/π_s_ already reported and would suggest that purifying selection is stronger in female‐biased genes.

### 3.8 Female ovary‐biased and gut‐biased genes

Next, we explored the expression of genes in female reproductive tissue. Overall there were a greater number of genes with gut‐biased expression (#Gut_Auto_ = 153) than ovary‐biased expression (#Ovary_Auto_ = 40) in the autosomes. However, there was an over‐representation of Z ovary‐expressed genes than expected by chance (#Gut_Z_ = 6; #Ovary_Z_ = 6; chi‐square test; *p *<* *0.05). However, the number of genes in each category is relatively small so these tests should be treated with caution.

Of the 205 differentially expressed genes between the two tissues, only 9 in the ovaries and 26 in the gut could be used to calculate dN/dS, π_n_/π_s_ and α. The other genes either do not have a 1‐1 ortholog with *H. erato* or there were too many undetermined characters (gaps or Ns) to be able to estimate the parameters. Of the 35 genes for which molecular evolution statistics could be calculated, all 9 ovary‐biased and 25 of 26 gut‐biased genes are autosomal; and 1 gut‐biased gene maps to the Z (Gut dN/dS _Z_ = 0.365; Gut π_n_/π_s__Z_ = 0.093; Gut α _Z_ = 0.295). We did not detect any significant differences in π_n_/π_s_; dN/dS; or α for autosomal ovary‐ and gut‐biased genes.

## 4 DISCUSSION

Elevated rates of coding sequence evolution on the sex chromosome relative to autosomes have been reported for several species, consistent with the theoretical prediction of fast‐X evolution. Here, we find evidence for enhanced rates of adaptation on the *Heliconius* Z chromosome: Z genes have a significantly higher rate of adaptive evolution when all expressed genes are considered. However, fast‐X theory predicts that genes highly expressed in the hemizygous sex should be especially prone to fast‐X evolution, and this prediction was not satisfied in our data. Female‐biased genes did not evolve faster when located on the Z chromosome. The evidence for fast‐Z evolution in *Heliconius* is, therefore, mixed.

In other taxa, there is strongest support for fast‐X evolution in groups with complete dosage compensation (Mank, Vicoso, et al., 2010; Meisel & Connallon, 2013). Theory predicts that opportunities for fast‐X evolution should increase in species with somatic X‐inactivation such as eutherian mammals, as there is effectively haploid expression of the sex chromosome in cells, increasing the chances of recessive beneficial mutations being fixed (Charlesworth, 2012). Groups such as Lepidoptera have been reported to have more complex patterns of sex chromosome dosage compensation. In *Heliconius* males, expression of Z genes is reduced below autosomal levels, but this dosage compensation mechanism is imperfect, with males showing increased expression relative to females on Z chromosome genes (Walters et al., 2015). However, the apparent incomplete dosage compensation could be a consequence of an uneven distribution of sex‐biased genes on sex chromosomes (Gu & Walters, 2017, Huylmans, Macon, & Vicoso, 2017). Regardless, when we compare rates of divergence and adaptation for genes with sex‐biased expression, the expectations of fast‐Z evolution are not clearly met.

Although we might expect faster rates of adaptive evolution for female‐biased genes, we observe a weak tendency for faster rates of evolution in male‐biased genes. This might mean that the Z could in fact be a hotspot for dominant alleles that benefit males. If this is the case, faster rates of evolution in male Z genes could contribute to a fast‐Z effect that is unrelated to hemizygous nature of the Z in females (Rice, 1984). It is important to note that sex‐specific genes can evolve rapidly both due to (a) neutral processes (as they experience relaxed selection in the other sex) and (b) the accumulation moderately deleterious mutations (Dapper & Wade, 2016; Gershoni & Pietrokovski, 2014, 2017; Mank, 2017). Nevertheless, our results of ω_α_ for male‐biased Z‐linked genes seem to support faster adaptive evolution of such genes: as the Z spends less time in females as compared to autosomes, relaxed selection should be greater for autosomal male‐biased genes (Rice, 1984).

Although a fast‐Z effect has been observed in *Bombyx mori* (Sackton et al., 2014), no such pattern was reported in two satyrine butterflies where the dN/dS ratio of Z genes was slightly lower than for autosomal genes (Rousselle et al., 2016). In *Heliconius*, although dN/dS was not significantly different between autosomal and Z genes, we did find evidence for a faster rate of adaptive substitution. Interestingly, our data also show that dS on the Z chromosome is higher than dS on the autosomes perhaps indicating a male‐biased mutation rate, as Z chromosomes spend more time in males than in females (Miyata, Hayashida, Kuma, Mitsuyasu, & Yasunaga, 1987). Although hemizygosity is expected to expose beneficial mutations to selection and increase rates of adaptive evolution on the Z chromosome, it is also expected to increase the efficacy of purifying selection, which would act to reduce evolutionary rates. It may be that the balance between these two forces differs across lepidopteran species, leading to the mixed pattern of fast‐Z evolution in some taxa but not others. It is important to add, however, that the α values estimated in this study are substantially higher than α reported in Martin et al. (2016). Martin et al. (2016) estimated α using the approach developed by Messer and Petrov (2013), and, in simulations, it has been shown that it is possible that there is an overestimation of DFE‐α (the method used in this study) in scenarios with strong sweeps or population expansion (Messer & Petrov, 2013).

Wright et al. (2015) interpreted the high dN/dS in Z genes of birds as a consequence of reduced effective population size rather than positive selection. The difference in effective population size between sex chromosomes and autosomes in female heterogametic systems is predicted to be larger than in male heterogametic systems due to higher variance of male reproductive success (Mank, Nam et al., 2010). Indeed, we estimate that coding regions on the Z chromosome have an *N*_e_ 0.44 times that of autosomes. We might therefore predict a considerable reduction in the efficacy of purifying selection on butterfly Z chromosomes. This should lead to higher ω_na_ and π_n_/π_s_ ratios on the Z due to stronger genetic drift. However, as in satyrine butterflies (Rousselle et al., 2016), in *Heliconius*, ω_na_ is not higher on the Z relative to autosomes. In *Heliconius*, dN/dS and π_n_/π_s_ are higher in the Z compared to autosomes, but this is not significant. This means that, in contrast to birds, the difference in the effective population size of the Z relative to autosomes is not sufficient to reduce the efficacy of purifying selection at a detectable level.

One possible explanation for this difference is the generally much higher effective population sizes of Lepidoptera, which could allow for efficient selection even on sex chromosome (Rousselle et al., 2016). Another is that by not using all genomic sites to estimate the π_sZ_/π_sA_ diversity ratio, we might be underestimating its true value due to a stronger effect of background selection. The latter is supported by the observation that, in a recently published paper using all genomic sites to estimate π_sZ_/π_sA_, Van Belleghem et al. (2018) calculated it to be 0.661 CI = [0.570–0.653]. Regardless, both ours and Van Belleghem et al. (2018) estimates of the π_sZ_/π_sA_ diversity ratio are significantly lower than the expected 0.75, and there is no observable reduction in the efficacy of purifying selection in *H. melpomene*.

Another factor that might counteract the fast‐Z effect is adaptation from standing variation. Larger populations are more polymorphic and, therefore, have an increased probability of adaption from standing genetic variation. Adaptation from standing genetic variation is expected to result in faster autosome evolution, independent of the dominance of beneficial alleles (Orr & Betancourt, 2001), which would counteract the fast‐Z effect. This may be especially relevant when overall population sizes are large, as in many *Heliconius* species, such that standing variation becomes a comparatively important source of adaptive variation compared to de novo mutations.

As sex genes tend to be expressed in sex‐specific tissue such as the testis and the ovaries, we aimed to investigate patterns of molecular evolution in ovary‐biased genes. Unfortunately, there are no ovary‐biased genes with 1‐1 orthologs between *H. melpomene* and *H. erato* that are in the Z. This means we could not test the effect of hemizygosity on non‐sex‐specific and sex‐specific female expression directly. The lack of 1‐1 orthology may mean that these genes are rapidly evolving. On the one hand, adaptive evolution rates could be higher. And, indeed, autosomal ovary‐expressed genes have higher rates of adaptive evolution than gut expressed genes. On the other hand, the lack of 1‐1 orthology could also be due to rapid nonadaptive evolution (Gershoni & Pietrokovski, 2014).

It has been recently shown that, like in other taxa, sex‐biased genes can experience rapid turnovers in butterflies (e.g., *Papilio*) (Huylmans et al., 2017). Consequently, it is possible for *H. melpomene* and *H. erato* to have different sex‐biased genes. However, if there are differences in sex‐biased genes between *H. melpomene* and *H. erato*, these are likely to only occur in small number of genes. *H. melpomene* and *H. erato* diverged 12MYA (Kozak et al., 2015) compared to 35MYA for *P. xuthus* and *P. machaon* (Zakharov, Caterino, & Sperling, 2004). Regardless, any gene that is sex biased in *H. erato* but not in *H. melpomene* is categorized as unbiased in our data. So, for example, if a fast‐evolving Z gene is female biased in *H. erato*, but unbiased in *H. melpomene*, the divergence and adaptation estimates could be inflated for the *Unbiased* category. In the future, analysing gene expression data from *H. erato* to understand whether sex‐biased genes are different for the two species would contribute to understand the actual gene turnover.

Together these results illustrate the need to study substitution rates in other ZW systems considering sex‐biased expression. This genomewide analysis of polymorphism, divergence and gene expression data contributes to a growing body of literature on sex chromosome evolution in ZW systems, and reveals the complexity of the different evolutionary forces shaping transcriptome evolution in *Heliconius* and, consistent with previous work, shows limited evidence of fast‐Z evolution in this taxon.

## Data Availability

Sequencing data are available from the European Nucleotide Archive (http://www.ebi.ac.uk/ena PRJEB30552). Data files can be found in the Dryad repository associated with this manuscript (https://doi.org/10.5061/dryad.51rk4v4).
