# Supplementary material for: Sexually dimorphic gene expression and transcriptome evolution provide mixed evidence for a fast‐Z effect in Heliconius
Source: J Evol Biol. 2019 Jan 11;32(3):194–204. doi: 10.1111/jeb.13410 (PMC6850379; doi:10.1111/jeb.13410)
Supplement: Supplementary file 1 [file JEB-32-194-s001.docx]

**Supplementary Tables**

- **Supplementary Table S1**. Sample information and statistics
- **Supplementary Table S2.** Orthologue prediction improvement with Hmel2.5 annotation
- **Supplementary Table S3.** Mean and median read-depth for resequenced whole-genome *H. melpomene* samples (van Belleghem *et al.*, 2018)

**Supplementary Table S1. Sample information and statistics**

| **Sample** | **Sex** | **Tissue** | **Treatment** | **Library** | **Raw Reads** |
| --- | --- | --- | --- | --- | --- |
| AP141 | Female | Gut | Old | RRB03031 | 32306468 |
| AP93 | Female | Gut | Young | RRB03025 | 34353682 |
| AP142 | Female | Gut | Old | RRB03032 | 34445514 |
| AP77 | Female | Gut | Old | RRB03030 | 32024898 |
| AP89 | Female | Gut | Old | RRB03034 | 39661158 |
| AP55 | Female | Gut | Old | RRB03035 | 35862378 |
| AP94 | Female | Gut | Young | RRB03026 | 36223403 |
| AP37 | Female | Gut | Young | RRB03029 | 31187077 |
| AP34 | Female | Gut | Young | RRB03028 | 35452206 |
| AP71 | Female | Gut | Young | RRB03024 | 33088963 |
| AP35 | Female | Gut | Young | RRB03027 | 46122142 |
| AP80 | Female | Gut | Old | RRB03033 | 40040750 |
| AP35 | Female | Ovaries | Young | RRBL00006 | 35018481 |
| AP94 | Female | Ovaries | Young | RRB02962 | 39903075 |
| AP34 | Female | Ovaries | Young | RRB02963 | 27811550 |
| AP37 | Female | Ovaries | Young | RRB02960 | 33410348 |
| AP71 | Female | Ovaries | Young | RRBL00007 | 34856038 |
| AP88 | Female | Ovaries | Young | RRBL00008 | 38486006 |
| AP93 | Female | Ovaries | Young | RRB02961 | 31497198 |
| AP55 | Female | Ovaries | Old | RRB03012 | 37934077 |
| AP77 | Female | Ovaries | Old | RRB03013 | 34322656 |
| AP80 | Female | Ovaries | Old | RRB03014 | 36157750 |
| AP89 | Female | Ovaries | Old | RRB03015 | 34318423 |
| AP141 | Female | Ovaries | Old | RRB03016 | 33844256 |
| AP142 | Female | Ovaries | Old | RRB03017 | 35328097 |
| R20 | Female | Abdomen | Young | NA | NA |
| R29 | Female | Abdomen | Young | NA | NA |
| R06 | Male | Abdomen | Young | NA | NA |
| R32 | Male | Abdomen | Young | NA | NA |
| R34 | Male | Abdomen | Young | NA | NA |
| R07 | Female | Abdomen | Young | NA | NA |
| R33 | Male | Abdomen | Young | NA | NA |
| R05 | Female | Abdomen | Young | NA | NA |
| R21 | Male | Abdomen | Young | NA | NA |
| R28 | Female | Abdomen | Young | NA | NA |

**Supplementary Table S1. Sample information and statistics (cont.)**

| **Sample** | **Raw Base (G)** | **Error Rate (%)** | **Q20 (%)** | **Q30 (%)** | **GC content (%)** |
| --- | --- | --- | --- | --- | --- |
| AP141 | 9.69 | 0.01 | 98.25 | 95.7 | 41.52 |
| AP93 | 10.31 | 0.01 | 97.38 | 93.98 | 40.89 |
| AP142 | 10.33 | 0.01 | 98.25 | 95.75 | 41.5 |
| AP77 | 9.61 | 0.01 | 98.23 | 95.69 | 42.14 |
| AP89 | 11.9 | 0.01 | 98.11 | 95.53 | 42.53 |
| AP55 | 10.76 | 0.01 | 98.15 | 95.6 | 41.52 |
| AP94 | 10.87 | 0.01 | 97.94 | 95.03 | 41.41 |
| AP37 | 9.36 | 0.01 | 98.52 | 96.42 | 42.95 |
| AP34 | 10.64 | 0.01 | 98.53 | 96.4 | 41.49 |
| AP71 | 9.93 | 0.01 | 98.12 | 95.39 | 40.62 |
| AP35 | 13.84 | 0.01 | 97.84 | 94.81 | 40.47 |
| AP80 | 12.01 | 0.01 | 98.19 | 95.62 | 42 |
| AP35 | 10.51 | 0.01 | 98.53 | 96.41 | 41.08 |
| AP94 | 11.97 | 0.01 | 98.16 | 95.19 | 41.51 |
| AP34 | 8.34 | 0.01 | 98.2 | 95.27 | 41.42 |
| AP37 | 10.02 | 0.01 | 98.49 | 95.88 | 42.15 |
| AP71 | 10.46 | 0.01 | 98.42 | 96.19 | 41.36 |
| AP88 | 11.55 | 0.01 | 98.53 | 96.37 | 42.71 |
| AP93 | 9.45 | 0.01 | 98.38 | 95.69 | 41.24 |
| AP55 | 11.38 | 0.01 | 98.17 | 95.51 | 39.76 |
| AP77 | 10.3 | 0.01 | 98.28 | 95.6 | 40.46 |
| AP80 | 10.85 | 0.01 | 97.97 | 95.06 | 40.28 |
| AP89 | 10.3 | 0.01 | 98.04 | 95.16 | 41.51 |
| AP141 | 10.15 | 0.01 | 97.88 | 94.83 | 39.58 |
| AP142 | 10.6 | 0.01 | 98 | 95.09 | 39.69 |
| R20 | NA | NA | NA | NA | NA |
| R29 | NA | NA | NA | NA | NA |
| R06 | NA | NA | NA | NA | NA |
| R32 | NA | NA | NA | NA | NA |
| R34 | NA | NA | NA | NA | NA |
| R07 | NA | NA | NA | NA | NA |
| R33 | NA | NA | NA | NA | NA |
| R05 | NA | NA | NA | NA | NA |
| R21 | NA | NA | NA | NA | NA |
| R28 | NA | NA | NA | NA | NA |

**Supplementary Table S1. Sample information and statistics (cont.)**

| **Sample** | **Mapped Reads(%)** | **Properly Paired(%)** |
| --- | --- | --- |
| AP141 | 83.40 | 78.16 |
| AP93 | 75.27 | 69.38 |
| AP142 | 79.84 | 74.32 |
| AP77 | 79.71 | 74.03 |
| AP89 | 73.41 | 67.05 |
| AP55 | 77.95 | 72.08 |
| AP94 | 62.79 | 58.07 |
| AP37 | 76.98 | 72.13 |
| AP34 | 81.84 | 76.85 |
| AP71 | 79.86 | 74.15 |
| AP35 | 76.68 | 71.58 |
| AP80 | 75.17 | 69.39 |
| AP35 | 76.76 | 71.96 |
| AP94 | 82.03 | 77.30 |
| AP34 | 86.45 | 81.59 |
| AP37 | 83.17 | 78.42 |
| AP71 | 85.70 | 80.81 |
| AP88 | 86.99 | 82.85 |
| AP93 | 82.80 | 78.53 |
| AP55 | 85.29 | 80.51 |
| AP77 | 87.83 | 83.17 |
| AP80 | 86.56 | 81.74 |
| AP89 | 85.34 | 80.68 |
| AP141 | 88.09 | 83.44 |
| AP142 | 87.19 | 82.62 |
| R20 | 38.20 | 34.46 |
| R29 | 31.92 | 29.07 |
| R06 | 60.95 | 53.47 |
| R32 | 85.00 | 77.32 |
| R34 | 29.08 | 26.05 |
| R07 | 83.25 | 76.15 |
| R33 | 35.84 | 30.56 |
| R05 | 71.44 | 64.81 |
| R21 | 38.06 | 31.36 |
| R28 | 61.37 | 54.10 |

**Supplementary Table S1. Sample information and statistics**

*H. melpomene rosina* RNA-seq mapping statistics. Sample ID, species, tissue, stage of collection for mRNA 150bp PE directionally sequenced reads for this project. Samples mapped to *H. melpomene* genome v2.1. Walters *et al.* (2015) sample mapping statistics to *H. melpomene* genome v2.1.

**Supplementary Table S2. Orthologue prediction improvement with Hmel2.5 annotation**

| **Statistics** | Hmel2 | **Hmel2.5** |  |
| --- | --- | --- | --- |
| # genes | 33137 | 41779 |  |
| # genes in orthogroups | 24274 | 29698 |  |
| # unassigned genes | 8863 | 12081 |  |
| % genes in orthogroups | 73.3 | 71.1 |  |
| % unassigned genes | 26.7 | 28.9 |  |
| # orthogroups | 9320 | 11062 |  |
| # species-specific orthogroups | 15 | 18 |  |
| # genes in species-specific orthogroups | 56 | 105 |  |
| % genes in species-specific orthogroups | 0.2 | 0.3 |  |
| Mean orthogroup size | 2.6 | 2.7 |  |
| Median orthogroup size | 2.0 | 2.0 |  |
| G50 assigned genes | 2 | 2 |  |
| G50 all genes | 2 | 2 |  |
| O50 assigned genes | 3252 | 3638 |  |
| O50 all genes | 5468 | 6658 |  |
| # of orthogroups with all species present | 9305 | 11044 |  |
| # of single-copy orthogroups | 6846 | 8095 |  |

**Supplementary Table S2. Orthologue prediction improvement with Hmel2.5 annotation**

Statistics on orthologue prediction between *H. melpomene* v2 annotation and *H. erato* annotation; and on orthologue prediction between *H. melpomene* v2.5 annotation and *H. erato* annotation.

**Supplementary Table S3. Mean and median read depth (RD) for resequenced whole-genome *H. melpomene* samples (van Belleghem *et al.*, 2018)**

| **Sample** | **Species** | **Sex** | **Location** | **Mean RD** | **Median**  **RD** |
| --- | --- | --- | --- | --- | --- |
| CAM000531 | H. m. rosina | Male | 9º87’N 7º96’W | 30.59 | 29 |
| CAM000533 | H. m. rosina | Male | 9º87’N 7º96’W | 28.83 | 21 |
| CAM000546 | H. m. rosina | Male | 9º87’N 7º96’W | 27.47 | 26 |
| CAM001841 | H. m. rosina | Male | 9º87’N 7º96’W | 28 | 28 |
| CAM001880 | H. m. rosina | Male | 9º87’N 7º96’W | 22.76 | 23 |
| CAM002045 | H. m. rosina | Male | 9º87’N 7º96’W | 25.7 | 26 |
| CAM002059 | H. m. rosina | Male | 9º87’N 7º96’W | 36.77 | 32 |
| CAM002071 | H. m. rosina | Male | 9º87’N 7º96’W | 26.43 | 21 |
| CAM002519 | H. m. rosina | Male | 9º87’N 7º96’W | 26.68 | 22 |
| CAM002552 | H. m. rosina | Male | 9º87’N 7º96’W | 26.83 | 22 |

**Supplementary Table S3. Mean and median read depth (RD) for resequenced whole-genome *H. melpomene* samples**

*H. melpomene* resequenced samples mapped to Hmel2 genome using BWA-MEM

**Supplementary Figures**

- **Supplementary Figure S1.** Principal component analysis of gene expression profiles for the 10 whole abdomen male and female samples
- **Supplementary Figure S2.** π_n_ is negatively correlated to expression level
- **Supplementary Figure S3.** Principal component analysis of gene expression profiles of *H. melpomene* females for 13 ovary samples and 12 gut samples at two different time points
- **Supplementary Figure S4.** Principal component analysis of gene expression profiles of *H. melpomene* females for 13 ovary samples and 12 gut samples at two different time points separated by tissue

**Supplementary Figure S1. Principal component analysis of gene expression profiles for the 10 whole abdomen male and female samples**

**Supplementary Figure S1. Principal component analysis of gene expression profiles for the 10 whole abdomen male and female samples**

PCA of the abdomen transformed gene expression count data to the log2 scale (DESeq2, rlog(blind=FALSE)). rlog transformed data minimises differences between samples for rows with small counts and normalizes with respect to library size.

**Supplementary Figure S2. π_n_ is negatively correlated to expression level**

**A.**

**B.**

|  | **estimate** | **std. Error** | **value** | **Pr(>\|t\|)** |
| --- | --- | --- | --- | --- |
| (Intercept) | -4.318 | 0.06 | -71.617 | < 2e-16 |
| logpiS | 0.469 | 0.015 | 31.164 | < 2e-16 |
| chromosomesex | -0.226 | 0.071 | -3.174 | 0.002 |
| logRPKMi | -0.035 | 0.006 | -5.529 | 3.36e-08 |

Residual standard error: 1.008 on 5428 degrees of freedom

Multiple R-squared:  0.1828, Adjusted R-squared:  0.1824

F-statistic: 404.9 on 3 and 5428 DF, p-value: < 2.2e-16

**Supplementary Figure S2. π_n_ is negatively correlated to expression level**

Multiple regression approach shows that π_n_ was significantly negatively correlated to expression level – autosomal (*P* < 0.001) and Z genes (*P* < 0.01). **A.** Plotted *Residuals vs Fitted* shows spread residuals around the horizontal line without distinct patterns. *Normal Q-Q* follow a straight line with residuals well lined. The *Scale-Location* plot shows residuals spread equally around range of predictors. There is equal variance or homoscedasticity. *Residuals vs Leverage* plot does not identify any influential outliers in the linear regression analysis. **B.** Regression coefficient table. Relationship between π_n_ and expression.

**Supplementary Figure S3. Principal component analysis of gene expression profiles of *H. melpomene* females for 13 ovary samples and 12 gut samples at two different time points**

**Supplementary Figure S3. Principal component analysis of gene expression profiles of *H. melpomene* females for 13 ovary samples and 12 gut samples at two different time points**

PCA of the female ovary and gut transformed gene expression count data to the log2 scale (DESeq2, rlog(blind=FALSE)). rlog transformed data minimises differences between samples for rows with small counts and normalizes with respect to library size.

**Supplementary Figure S4. Principal component analysis of gene expression profiles of *H. melpomene* females for 13 ovary samples and 12 gut samples at two different time points separated by tissue**

**Supplementary Figure S4. Principal component analysis of gene expression profiles of *H. melpomene* females for 13 ovary samples and 12 gut samples at two different time points separated by tissue type**

PCA of the female ovary and gut transformed gene expression count data to the log2 scale (DESeq2, rlog(blind=FALSE)) separated by tissue. rlog transformed data minimises differences between samples for rows with small counts and normalizes with respect to library size. **A.** 45% of the variance is explained by PC1 and PC2. PC1 separates young ovary tissue from old ovary tissue and explains 29% of the variance. All the samples cluster by age. **B.** 39% of the total variance is explained by PC1 and PC2. PC1 separates young gut tissue from old gut tissue and explains 23% of the variance. The samples cluster less tightly by age than ovary expression.
